# Insulin Access Enhancement in India: Expert Views on Integrating Interchangeable Biosimilar Insulin Glargine

**DOI:** 10.7759/cureus.60983

**Published:** 2024-05-24

**Authors:** KM Prasanna Kumar, Subhankar Chowdhury, Ganapathi Bantwal, A G Unnikrishnan, Sanjay Kalra, Sameer Aggarwal, Awadhesh Kumar Singh, Kaushik Pandit, Rishi Shukla, Vijay Vishwanathan, Kunal Khobragade, Prashant S Sarda

**Affiliations:** 1 Department of Endocrinology, Center For Diabetes and Endocrine Care (CDEC), Bengaluru, IND; 2 Diabetes & Endocrinology, Medica Super Specialty Hospital, Kolkata, IND; 3 Endocrinology and Diabetes, St. John's Medical College Hospital, Bengaluru, IND; 4 Endocrinology, Chellaram Diabetes Institute, Pune, IND; 5 Endocrinology, Bharti Research Institute of Diabetes & Endocrinology (BRIDE), Karnal, IND; 6 Endocrinology, Apex Plus Superspeciality Hospital, Rohtak, IND; 7 Endocrinology, GD Hospital & Diabetes Institute, Kolkata, IND; 8 Endocrinology, Diabetes and Metabolism, Fortis Medical Centre, Kolkata, IND; 9 Endocrinology, Center For Diabetes and Endocrine Disease, Kanpur, IND; 10 Diabetology, M. Viswanathan (MV) Hospital for Diabetes, Chennai, IND; 11 Diabetology, Prof. M. Viswanathan Diabetes Research Centre, Chennai, IND; 12 Medical Affairs, Mankind Pharma Ltd., Navi Mumbai, IND

**Keywords:** reference insulin glargine, originator, diabetes, biosimilar insulin glargine-yfgn, adherence

## Abstract

Achieving and maintaining optimal glycemic targets is the fundamental goal of the management of diabetes. However, failure of oral antidiabetic drugs (OADs) to sustain the targeted glycemic levels in individuals with progressing disease often requires initiation of insulin therapy. This article consolidates the expert opinions of 377 doctors who participated in 34 advisory board meetings held digitally (n=23) and in person (n=11) across India. The present report underscores the need for readily available alternatives, such as biosimilar insulins, in the Indian healthcare market to make insulin accessible to every patient with diabetes. The introduction of biosimilar insulins in the Indian healthcare market is the key to making insulin accessible to every patient with diabetes. Biosimilars are biologic products that closely resemble reference/originator biologics and demonstrate no clinically meaningful differences in safety and effectiveness. The concept of interchangeability serves as a pivotal differentiator for biosimilars, underlining their reliability and safety, and plays a significant role in their broader acceptance and integration into healthcare systems. The 'interchangeability' designation by the United States Food and Drug Administration (USFDA) elevates the biosimilar concept, promoting faster and broader adoption of insulin biosimilars, especially benefiting patients prone to non-adherence to insulin therapy. Healthcare providers are encouraged to consider the option of initiating or transitioning to biosimilar insulin glargine to address the insulin accessibility challenges.

## Introduction and background

The treatment of type 2 diabetes mellitus (T2DM) primarily aims to reduce the risk of diabetes-related complications and subsequent diabetes-related mortality, by effectively achieving and maintaining glycemic control [1]. While initial glycemic control can be achieved using oral antidiabetic drugs (OADs), it might be challenging to attain the target levels in individuals with T2DM as the disease progresses, necessitating a combination of medications and the initiation of insulin therapy [2]. Poor glycemic control remains a persistent issue in India, even with at least two to three OADs being used. Early initiation of insulin therapy is crucial to achieve optimal glycemic control among Indians with uncontrolled T2DM.

Despite half of the world's population requiring this life-saving medication, many still lack adequate access to insulin which is often attributed to its high costs, limited availability of human insulin, few producers dominating the insulin market and weak health systems [3-5]. The worldwide transition from cost-effective human insulin to more expensive analogs (synthetic insulins) is placing an unsustainable financial strain on lower-income nations. Additionally, costing of insulin in the market is not transparent and biosimilar insulins, which essentially serve as generic alternatives, have the potential to be over 25% more cost-effective than the original product. Nevertheless, numerous countries, including those with lower incomes, are not capitalizing on this possible cost reduction.

In India, the absence of robust market competition, direct sales routes that circumvent pharmacies, and a shift toward pricier analog insulin are collectively erecting substantial obstacles to accessing insulin [6]. Additionally, it is worth noting that both doctors and patients in India have a prevailing perception that insulin from international companies is of higher quality, and they tend to be more skeptical of products from Indian companies [6]. Availability of insulin is better in private pharmacies and private hospitals, where people have to pay out of pocket [7]. Hence, the introduction of biosimilar insulins, an affordable alternative to analog insulins, in the Indian healthcare market is the key to making insulin accessible to every patient with diabetes.

Evidence supporting the use of biosimilar insulin in real-world practice across India is limited, and current recommendations for their use are based on level D evidence. Therefore, expert opinion from India is needed which will bridge the gap between randomized clinical trials and real-world evidence and provide practical guidance for clinicians regarding the clinical use of biosimilar insulins. The present article comprehensively describes how interchangeable biosimilar insulin glargine can be an effective solution for affordable and accessible insulin in India and presents the perspective of Indian experts on integrating interchangeable biosimilar insulin glargine in real-world clinical practice.

## Review

Methods

Advisory board meetings were conducted between May 2023 and July 2023 to collect expert opinions from across India on how interchangeable biosimilar insulin glargine can be an effective solution for accessible insulin in the country. A total of 34 advisory board meetings were conducted, including 23 digital and 11 physical meetings across India. Out of the 377 doctors included, 210 attended the digital meetings, while 167 were present at the physical meetings. Discussions in the meetings were focused on following aspects: i) Role of biosimilars in addressing insulin accessibility and regulatory considerations for adopting biosimilars; ii) equivalence of biosimilars to the reference product; iii) switch from the reference insulin glargine product to interchangeable biosimilar insulin glargine; iv) accessibility of interchangeable biosimilar insulin glargine in India; and v) overview of barriers before prescribing interchangeable biosimilar insulin.

Role of Biosimilars in Addressing Insulin Accessibility and Reliability of Physicians on USFDA-Approved Biosimilar

With the ongoing increase in diabetes prevalence in India, there is a growing demand for cost-effective biosimilar insulin options [8]. High number of diabetes patients struggling to manage their uncontrolled glycemia can be partially linked to the expensive nature of insulin. Biosimilar insulins are progressing toward global availability, offering a solution for all diabetes patients. Introduction of these biosimilar insulins to the market is expected to foster increased competition, which should ultimately result in reduced insulin prices for all patients [9]. Hence, the availability of biosimilar insulin can help to overcome the hurdle of insulin inaccessibility.

Several potential obstacles persist in the adoption of biosimilars [10]. There is a prevalent skepticism or mistrust regarding biosimilars, often linked to misconceptions about their efficacy, safety, or quality in comparison to originator biologics. Worries regarding the immunogenicity and safety profile of biosimilars may deter both healthcare professionals and patients from embracing these products. Questions regarding the interchangeability of biosimilars with existing insulins, including concerns about potential differences in clinical outcomes, may impede their widespread use. Hence, it is imperative to address these barriers through education, awareness, and robust clinical evidence to facilitate the adoption of biosimilars in healthcare settings.

**Figure 1:**
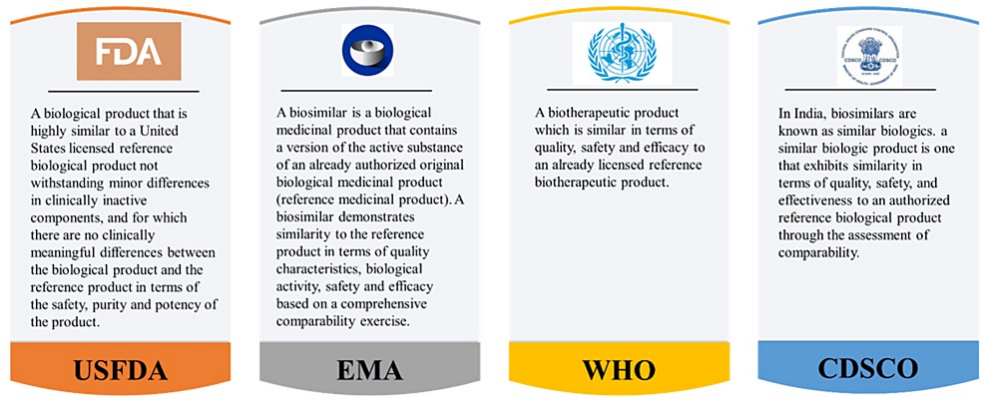
Definitions of Biosimilar CDSCO: Central Drugs Standard Control Organization; DBT: Department of Biotechnology; EMA: European Medicines Agency; FDA: Food and Drug Administration; WHO: World Health Organization This figure was drawn by the authors of this article.

A comprehensive regulatory framework is in place for the approval of biosimilars. Over the years, regulatory bodies in the USA and Europe have released scientific guidelines aimed at assisting developers in meeting the stringent regulatory standards for biosimilar approval. These guidelines have adapted to stay in alignment with the rapid advancements in biotechnology and analytical sciences, incorporating the growing wealth of clinical experience into their recommendations. While both biosimilars and reference medicines have rigorous data requirements [11], biosimilars often rely on comparative data to demonstrate similarity to the reference product, while reference medicines are subjected to full-scale clinical trials and extensive nonclinical testing to establish their safety and efficacy. The specific requirements may vary depending on the regulatory agency and the nature of the product. Figure 1 provides definitions of biosimilar according to different regulatory agencies [12-16]. Approval for medicines (original or biosimilar) is granted when rigorous assessments of their pharmaceutical quality, safety, and efficacy provide compelling evidence that the benefits of the medicine outweigh its potential risks, resulting in a favorable “positive benefit-risk” profile. In the case of biosimilars, a favorable benefit-risk assessment hinges on establishing biosimilarity, meaning that the active ingredient closely resembles that of the reference medicine. This is accomplished through thorough comparability studies with the reference medicine, supported by robust pharmaceutical quality data. Data requirements for biosimilars and new biological medicines differ because biosimilars primarily rely on demonstrating similarity to an existing reference product, while new biological medicines involve more extensive nonclinical and clinical testing to establish the safety and efficacy of a novel active substance. Biosimilar approval leverages the existing scientific knowledge regarding the safety and efficacy of the reference medicine, which has been acquired through its clinical use (Table 1). Consequently, the approval process for biosimilars typically requires a reduced amount of clinical data compared to entirely novel medications.

**Table 1:** Experts' opinion on the reliability of physicians on USFDA-approved biosimilar USFDA: United States food and drug administration

Experts’ perspectives on the reliability of physicians on USFDA-approved biosimilar
“Biosimilars are USFDA-approved products that have undergone rigorous evaluation to establish their similarity to the reference product in terms of structure, efficacy, and safety and hence there is no need to focus on safety data.”
“Physicians can confidently rely on USFDA-approved biosimilars, as well as interchangeable biosimilars, for patient treatment.”

Equivalence of Biosimilar Insulin Glargine-yfgn to Original Insulin Glargine

Robust evidence from two landmark clinical trials, INSTRIDE 1 and INSTRIDE 2, indicates that insulin glargine-yfgn exhibits the same pharmacodynamic and pharmacokinetic properties as the reference insulin glargine [17,18]. The INSTRIDE 1 trial was conducted to evaluate the efficacy and safety of biosimilar insulin glargine-yfgn (MYL-1501D) vs reference insulin glargine. Similarly, INSTRIDE 2 evaluated the efficacy and safety of biosimilar insulin glargine-yfgn vs reference insulin glargine but specifically included patients with T2DM who were on oral medications. Both studies established noninferiority of MYL-1501D to reference insulin glargine. Furthermore, both trials reported comparable immunogenicity profiles between biosimilar insulin glargine-yfgn and reference insulin glargine groups (Table 2). Overall, MYL-1501D was well tolerated, showing no new or significant safety issues compared to insulin glargine (Table 3). As a result of the INSTRIDE 1 and INSTRIDE 2 trials, insulin glargine-yfgn secured approval as a biosimilar in June 2020 [19].

**Table 2:** Experts’ opinion on equivalence of biosimilars to the reference product

Table *2*: Experts’ perspectives on equivalence of biosimilars to the reference product
“Biosimilar insulin glargine-yfgn holds significant promise as a viable alternative to the original insulin glargine, offering similar efficacy and safety profiles.”

**Table 3:** Overview of landmark trials on biosimilar insulin glargine AEs: adverse events; HbA1c: glycated hemoglobin; IG: insulin glargine; PD: pharmacodynamic; PK: pharmacokinetic; T1DM: type 1 diabetes mellitus

Study Characteristics	INSTRIDE 1	INSTRIDE 2	INSTRIDE 3 (Interchangeable Study)
Population	Type 1 diabetes (n = 558)	Type 2 diabetes (n = 560)	Type 1 diabetes (n = 127)
Study length (weeks)	52	24	52
Inclusion criteria	Patients on bolus (Lispro) + insulin glargine	Insulin-naive and on OADs	Patients on bolus (Lispro) + insulin glargine
Baseline HbA1c (Biosimilar vs Reference)	7.37% vs 7.39%	8.12 % vs 8.14%	7.60% vs 7.90%
Mean change in HbA1c (Biosimilar vs Reference)	- 0.21% vs – 0.25%	- 0.60% vs – 0.66%	Comparable difference
Hypoglycemia (Biosimilar vs Reference)	No statistically significant differences in hypoglycemic rate or incidence profiles between groups at any visit	No statistically significant differences in hypoglycemic rate or incidence profiles between groups at any visit	No significant difference observed between treatment sequences at any visit
Mean change in daily basal insulin dose (Biosimilar vs Reference)	0.0128 U/kg vs 0.0043 U/kg	0.24 U/kg vs 0.24 U/kg	Stable throughout the study
Conclusion	Bioequivalence of IG, US IG, and EU IG was demonstrated in patients with T1DM for both primary PK and PD endpoints.	Mean change in HbA1c from baseline to week 24 was within noninferiority margin of 0.4%.	Switching participants between interchangeable and reference insulin glargine demonstrated equivalent efficacy and similar safety and immunogenicity, showing that people taking reference insulin glargine can safely switch to interchangeable insulin glargine.
	Biosimilar IG, US IG, and EU IG were generally well tolerated, & no significant safety issues emerged	Rates of hypoglycemia and other AEs were comparable between the two groups.	

Switch from Reference Insulin Glargine Product to Interchangeable Biosimilar Insulin Glargine

The substantial financial burden of medication costs affects many adults with diabetes and leads to a decline in treatment adherence [20]. Therefore, implementing strategies that lower medication expenses can enhance treatment adherence in individuals with diabetes. Interchangeability at pharmacies can simplify the use of biosimilar insulin by streamlining the substitution process. Benefits include easier transition to a less expensive product, decreased time for medical provider approval, resulting in shorter waiting period at the pharmacy, and a smoother shift to lower-cost insulin, ultimately reducing healthcare system expenses [21].

The USFDA states that “availability of biosimilar and interchangeable products that meet the FDA’s robust approval standards will improve access to biological products through lower treatment costs and enable greater economies of scale in biosimilar manufacturing” [22]. The “interchangeability” designation by the USFDA elevates the biosimilar concept, promoting faster and broader adoption of insulin biosimilars, especially benefiting patients prone to non-adherence to insulin therapy [23].

**Figure 2:**
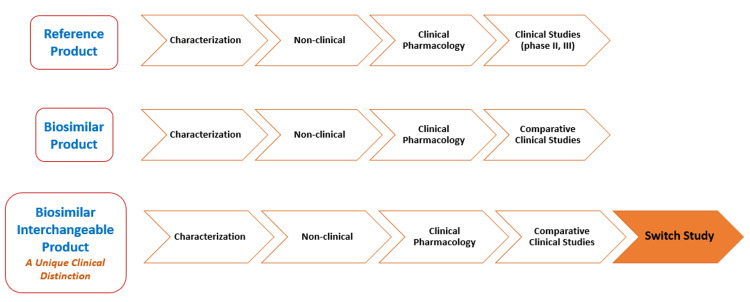
Regulatory Approval Pathway: Originator vs Biosimilar vs Interchangeable Biosimilar This figure was drawn by the authors of this article.

To achieve the FDA's classification as an interchangeable biosimilar, a biosimilar medication should undergo crossover or switch studies demonstrating equivalent clinical efficacy and safety outcomes to the reference biologic. Switch studies involve maintaining a control group on the reference medication while the intervention group undergoes alternating transitions between the reference medication and the biosimilar medication under investigation. Hence, an “interchangeable biosimilar insulin” is a type of biosimilar insulin that has been shown to produce the same clinical results with regard to efficacy and safety as the reference insulin, and it can be substituted for the reference insulin without the need for the healthcare provider's intervention. Figure 2 summarizes the regulatory approval pathways for originator/reference products, biosimilars, and interchangeable biosimilars.

Clinical studies that underpin the concept of interchangeability predominantly evaluate whether repetitive switching between a reference product and its biosimilar affects clinical effectiveness and whether such transitions lead to variances in pharmacokinetics or immunogenicity profiles [24,25]. From a clinical viewpoint, the probability of encountering risks when transitioning between a biologic reference product and its biosimilar is extremely low [24]. Furthermore, compelling evidence derived from interchangeability studies can instill a sense of trust in patients regarding these biosimilars. Consequently, patients can have the same level of confidence in the safety of these products as they would in the reference medicine (Table 4).

**Table 4:** Experts’ opinion on switch from reference insulin glargine product to interchangeable biosimilar insulin glargine

Experts’ perspectives on the switch from reference insulin glargine product to interchangeable biosimilar insulin glargine
“Individuals using the reference insulin glargine can make a safe and effective switch to the biosimilar insulin glargine.”

**Figure 3:**
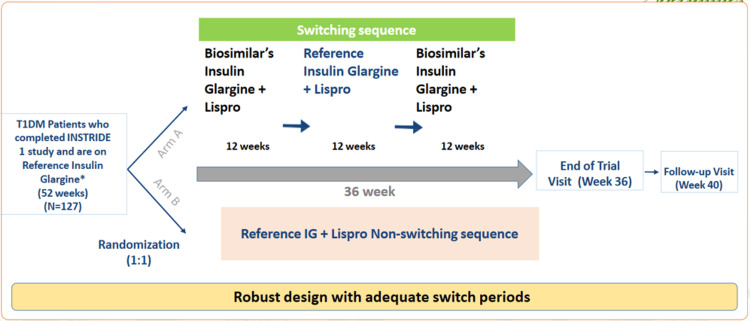
Robust Design with Adequate Switch Periods of INSTRIDE 3 Study This figure was drawn by the authors of this article.

The INSTRIDE 3 trial [26] represents an example of a switching study with robust design with adequate switch periods carried out in individuals with T1DM (Figure 3). Its primary objective was to evaluate the effectiveness, required insulin dosage, safety, and immunogenicity when patients transitioned between biosimilar insulin glargine-yfgn and the reference insulin glargine. Participants who had successfully completed the reference insulin glargine segment in INSTRIDE 1 were subject to randomization. They were either assigned to the reference insulin glargine group or the treatment-switching group. The latter received insulin glargine-yfgn for weeks 0 to 12, followed by reference insulin glargine for weeks 12 to 24, and then returned to insulin glargine-yfgn for weeks 24 to 36. The process of transitioning participants from reference insulin glargine to biosimilar insulin glargine-yfgn revealed equivalent efficacy, safety and immunogenicity profiles, indicating that individuals taking reference insulin glargine can safely switch to biosimilar insulin glargine-yfgn [26] (Table 3). After the results of the INSTRIDE 3 trial, it was subsequently awarded interchangeable biosimilar status with reference to insulin glargine in July 2021 [27].

Accessibility of Interchangeable Biosimilar Insulin Glargine in India

Interchangeable biosimilar insulin glargine is a safe option for switching from reference insulin glargine product. The process of substitution can manifest as a physician-guided substitution, often referred to as “switching,” which is commonly practiced in various member states of the EU, India, Japan, and across the globe [23]. Interchangeable biosimilar insulin glargine offers a pathway to access high-quality biologic therapy that would otherwise be unattainable due to its prohibitive cost. Designating biosimilars as “interchangeable” can stimulate a more rapid and widespread adoption of these insulin products, while also effectively managing insulin expenses. This is particularly crucial for patients who might otherwise resort to non-adherence or rationing of insulin, a life-saving medication. Potential future developments in insulin markets should encompass efforts aimed at enhancing the accessibility of interchangeable biosimilar insulin throughout tier 1, 2, 3, and 4 cities in India (Table 5). Promoting education, providing support, and increasing awareness are essential factors in fostering broader acceptance of interchangeable biosimilars.

**Table 5:** Experts’ opinion on accessibility of interchangeable biosimilar insulin glargine in India

Experts’ perspectives on accessibility of interchangeable biosimilar insulin glargine in India
“Interchangeable biosimilar insulin will help to improve the accessibility of insulin, a life-saving medicine, in India.”

Place of insulin glargine in clinical practice

American Diabetes Association recommends initiation of basal insulin as a first choice to achieve target fasting plasma glucose (FPG) levels in individuals with uncontrolled glycated hemoglobin (HbA1c) following >3 months of triple combination therapy, those with an HbA1c >10%, those with blood glucose levels >300 mg/dL, and those exhibiting symptoms of hyperglycemia or catabolic events [28]. According to the Research Society for the Study of Diabetes in India (RSSDI), the basal insulin dosage is estimated based on weight and is normally initiated at 10 U/day or 0.1 to 0.2 U/kg/day for individuals with an HbA1c <8%. For those with an HbA1c >8%, the recommended dosage is 0.2 to 0.3 U/kg/day [29,30]. A substantial increase in the adoption of basal insulin has been observed over the past decade, underpinning a significant paradigm shift in favor of greater basal insulin utilization across the country [31-34]. A steadily expanding body of evidence has allowed insulin glargine to address and alleviate the commonly held concerns linked to the utilization of basal insulin for managing diabetes [35-39]. Insulin glargine has brought about a transformative shift in the practices related to basal insulin usage in India. Furthermore, insulin glargine has proven its effectiveness and safety in unique circumstances and among special populations, including pregnant individuals, those with chronic kidney disease, and the elderly. Based on this substantial body of supporting evidence, various medical guidelines [29,40-44] also advocate for the utilization of insulin glargine due to its ability to provide safe and efficient glycemic control, especially in challenging scenarios and high-risk patient populations [45].

Simultaneously, data from a range of clinical studies consistently shows that biosimilar insulin glargine effectively lowers the risk of hypoglycemia and minimizes glycemic fluctuations, along with improvements in FPG, postprandial glucose, and HbA1c levels. Individuals who experience hypoglycemia are at an increased risk of developing cardiac arrhythmias, myocardial ischemia and facing a higher mortality rate [46,47]. Therefore, a reduced risk of hypoglycemia with the use of biosimilar insulin glargine may aid in reducing the risk of ischemia and arrhythmia. The low risk of hypoglycemia associated with insulin glargine is also beneficial for elderly patients [38,45]. Hence, a cost-efficient substitute like biosimilar insulin glargine-yfgn holds significant promise as a beneficial treatment choice for elderly patients in India. This is particularly relevant in light of the absence of medical insurance coverage for diabetes-related costs, restricted financial resources, or complete financial reliance on family support.

Role of a physician in the initiation of insulin

While the advantages of commencing insulin therapy early are evident, a substantial practical disparity exists in the initiation of insulin treatment among healthcare practitioners across India [48]. Barriers faced by physicians in commencing insulin therapy encompass factors such as insufficient knowledge, training, and experience, language barriers between physicians and patients, heightened physician apprehension regarding the risk of hypoglycemia and weight gain, inadequacy of physicians' skills, and the time necessary for insulin therapy, their perception of potential complications, a perceived lack of treatment benefits as well as concerns regarding patient noncompliance [48,49]. Nevertheless, it's crucial to recognize that physicians have a pivotal role to fulfill in the initiation of insulin therapy for effectively managing patients with poorly controlled diabetes. Physicians should motivate the patients to consider the use of basal insulin, emphasizing the advantages of its once-daily dosing during their interactions with patients. Moreover, the utilization of biosimilar insulin glargine is often contingent on the socioeconomic status of patients, and efforts should be made to overcome any inertia on both the physician's and patient's side in this regard. Commencing insulin treatment in insulin-naive patients should be accompanied by thorough counseling regarding the advantages of early initiation and the risks associated with delayed commencement.

Overview of Barriers Before Prescribing an Interchangeable Biosimilar Insulin

There are a few key aspects for overcoming barriers when considering the prescription of interchangeable biosimilar insulin. It is important to provide clear and detailed information to patients about the benefits and safety of interchangeable biosimilar insulin, addressing any concerns or misconceptions. Patient education on the proper and safe use of insulin pens is a critical component in enhancing adherence to biosimilar insulin glargine. It is imperative to provide patients with guidance on the correct injection technique to ensure safe and effective insulin administration. Cost plays a pivotal role in enhancing the accessibility of biosimilar insulin glargine. Even a slight reduction in the price of biosimilar insulin glargine can facilitate its introduction to a larger number of new patients. Pharmaceutical companies can extend their reach to rural areas of India effectively by leveraging their extensive sales force. This outreach effort has the potential to contribute to the wider adoption of biosimilar insulin glargine in India. Generation of real-world evidence and patient outcomes related to the use of interchangeable biosimilars will aid in building confidence among patients and healthcare providers in their effectiveness and safety. Storing insulin presents a dilemma in numerous resource-constrained environments, where reliable refrigeration may not consistently be at hand mainly due to the lack of electricity, literacy rate, and extreme temperatures [50,51]. Notably, in rural areas of India, the accessibility of insulin relies on the dependable provision of cold chain maintenance. Therefore, cold chain maintenance is a critical aspect of insulin storage and transportation (Table 6).

**Table 6:** Experts’ opinion on barriers to prescribing an interchangeable biosimilar insulin

Experts’ perspectives on barriers to prescribing an interchangeable biosimilar insulin
“Providing clear and detailed information about the benefits and safety of interchangeable biosimilar insulin to patients and addressing concerns and misconceptions will improve adherence and outcomes in diabetes management.”
“Pharmaceutical companies can effectively extend their reach to rural areas of India, potentially contributing to the broader adoption of biosimilar insulin glargine in the country.”
“Maintaining the cold chain is essential for storing and transporting biosimilar insulin glargine.”
“To ensure insulin is widely available, efforts should extend beyond metropolitan areas to include urban and rural regions. Distributing ice-lined refrigerators to rural pharmacies is vital for preserving the cold chain integrity of insulin.”
“Cold chain distribution, from the storage facility to the patient, should be diligently maintained. Each unit should receive a cold pack for distribution.”

Ensuring long-term availability and usage of insulin glargine is easily achievable by storing it in cold maintenance. The cold chain process is implemented to maintain medications within a specified temperature range, typically between 2 and 8°C, throughout the entire supply chain. Deviations from this recommended range can compromise the effectiveness of biologicals, resulting in an inadequate response [52,53]. Therefore, it is essential to employ a cold chain during the transportation of insulin from the production facility to the distributor's storage facility. Throughout the entire transport, a temperature log should be utilized. Upon receiving the insulin, the recipient should examine the temperature log and verify that the insulin has been delivered under proper cold chain conditions [52,53].

**Figure 4:**
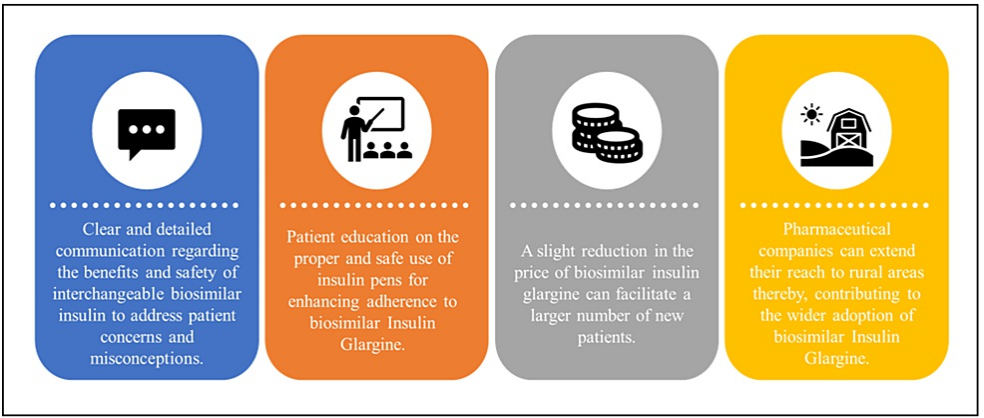
Barriers in the Prescription of Interchangeable Biosimilar Insulin This figure was drawn by the authors of this article.

All of the aforementioned aspects should be taken into account when addressing barriers in the prescription of interchangeable biosimilar insulin (Figure 4).

## Conclusions

While accessibility and affordability of insulin pose significant challenges in India, particularly in tier 2, tier 3, and tier 4 cities, 377 Indian experts opined that the interchangeable biosimilar insulin glargine is equally efficacious and safe to the reference insulin glargine and a viable solution which could greatly enhance the accessibility and affordability of insulin, a crucial medicine for managing diabetes, in real-world clinical practice. Future developments in insulin markets should focus on enhancing the accessibility of interchangeable biosimilar insulin across all tiers of cities in India, including tier 1, 2, 3, and 4 cities.
